# A novel approach for rapid high-throughput selection of recombinant functional rat monoclonal antibodies

**DOI:** 10.1186/s12865-018-0274-8

**Published:** 2018-12-04

**Authors:** Qin Chen, Shengping Qiu, Huanhuan Li, Chaolong Lin, Yong Luo, Wenfeng Ren, Yidi Zou, Yale Wang, Ninghshao Xia, Chenghao Huang

**Affiliations:** State Key Laboratory of Molecular Vaccinology and Molecular Diagnostics, National Institute of Diagnostics and Vaccine Development in Infectious Diseases, School of Public Health, Xiamen University, Xiamen, 361102 China

**Keywords:** Rat monoclonal antibody, Multiplex immunization, High-throughput, Recombinant expression, Immunotherapy study

## Abstract

**Background:**

Most monoclonal antibodies against mouse antigens have been derived from rat spleen-mouse myeloma fusions, which are valuable tools for purposes ranging from general laboratory reagents to therapeutic drugs, and yet selecting and expressing them remains a time-consuming and inefficient process. Here, we report a novel approach for the rapid high-throughput selection and expression of recombinant functional rat monoclonal antibodies with different isotypes.

**Results:**

We have developed a robust system for generating rat monoclonal antibodies through several processes involving simultaneously immunizing rats with three different antigens expressing in a mixed cell pools, preparing hybridoma cell pools, in vitro screening and subsequent cloning of the rearranged light and heavy chains into a single expression plasmid using a highly efficient assembly method, which can decrease the time and effort required by multiple immunizations and fusions, traditional clonal selection and expression methods. Using this system, we successfully selected several rat monoclonal antibodies with different IgG isotypes specifically targeting the mouse PD-1, LAG-3 or AFP protein from a single fusion. We applied these recombinant anti-PD-1 monoclonal antibodies (32D6) in immunotherapy for therapeutic purposes that produced the expected results.

**Conclusions:**

This method can be used to facilitate an increased throughput of the entire process from multiplex immunization to acquisition of functional rat monoclonal antibodies and facilitate their expression and feasibility using a single plasmid.

**Electronic supplementary material:**

The online version of this article (10.1186/s12865-018-0274-8) contains supplementary material, which is available to authorized users.

## Background

Mice are generally used to generate hybridoma cells producing monoclonal antibodies (mAbs) against antigens from various species but are seldom used to produce antibodies against mouse antigens due to their tolerance of syngeneic antigens [1, 2]. Rats can provide a large number of spleen B cells that are available for fusion with myeloma cells, which are extremely suitable for generating mAbs against mouse antigens [3]. The ability of rat monoclonal antibodies (RtmAbs) to bind with high selectivity and affinity to their targets makes them extremely important tools for biomedical research, especially for immune-detection of antigens from a mouse background and for functional evaluation of immunotherapeutic antibodies in immunocompetent mice [4–6]. In addition, RtmAbs possess great performance in recognizing additional epitopes, especially for small and poor immunogenic antigens [7, 8].

The cell fusion technique first reported in 1975 made it possible to generate hybridoma cells producing mAbs [9]. Since then, the methods for mAb production have been improved, and hybridoma technology is now well established [9, 10]. Most RtmAbs have been derived from rat spleen-mouse myeloma fusions using classical hybridoma technology [3, 11]. However, to our knowledge, some of the rat×mouse hybridoma clones grow slowly and would be gradually lost during the clonal selection step due to their instability, and it’s also difficult to produce high-quality or large scale RtmAbs from ascites or culture supernatants. Therefore, clonal selecting and expressing RtmAbs requires multiple rounds of clonal selection and continued cell culture, which makes it a time-consuming and inefficient process to obtain the desired RtmAbs [12].

Our aim was to perform rapid high-throughput selection and expression of functional RtmAbs with high affinity and specificity to mouse antigens that could be used as therapeutic mAbs in immunocompetent mouse models and for detecting murine antigens out of a mouse background. To achieve the high-throughput selection of antibodies of a given specificity, we have chosen the injection of pooled cells consisting of three different antigen-expressing cells, which are highly immunogenic and are simple to obtain and use [13]. This procedure may bring about a disadvantage in that the hybridoma cells will be producing antibodies against virtually all cellular antigens and will require several screening steps to select antigen-specific hybridoma cells; however, because only one cycle of screening is needed, this immunization protocol is easy, and multiple immunogens can be easily obtained, which can facilitate the high-throughput immunization process. Moreover, the multiplex immunization strategy, which simultaneously immunised the rat with three different antigens for the purpose of saving time and biological resources, has not been previously reported.

The biggest bottleneck in the screening process of stable and homogenous hybridoma clones is the time-consuming and labour-intensive screening of positive hybridoma cells. To streamline the screening process and avoid the clones missing, these steps were replaced by cloning the rearranged light and heavy chains into a single expression plasmid utilizing the highly efficient Gibson assembly method [14].

Here, we report a robust system for high-throughput generation of recombinant functional RtmAbs through several processes involving simultaneously immunizing rats with three different antigens-of-interest expressing in a mixed cell pools, preparing hybridoma cell pools, in vitro screening of colonized cells using high-throughput cell-based ELISA and subsequently cloning the rearranged light and heavy chains into a single expression plasmid using a highly efficient assembly method, which can successfully select several RtmAbs with different IgG isotypes targeting the mouse PD-1, LAG-3 or AFP protein. We applied the resulting recombinant anti-PD-1 antibodies in immunotherapy to therapeutic purposes that generated the expected results. This method can be used to facilitate an increased throughput and rapid process of enabling the generation of diverse panels of functional recombinant RtmAbs from immunized rats and other hosts.

## Results

Overview of a novel approach for the rapid high-throughput selection and expression of recombinant functional RtmAbs.

**Fig. 1:**
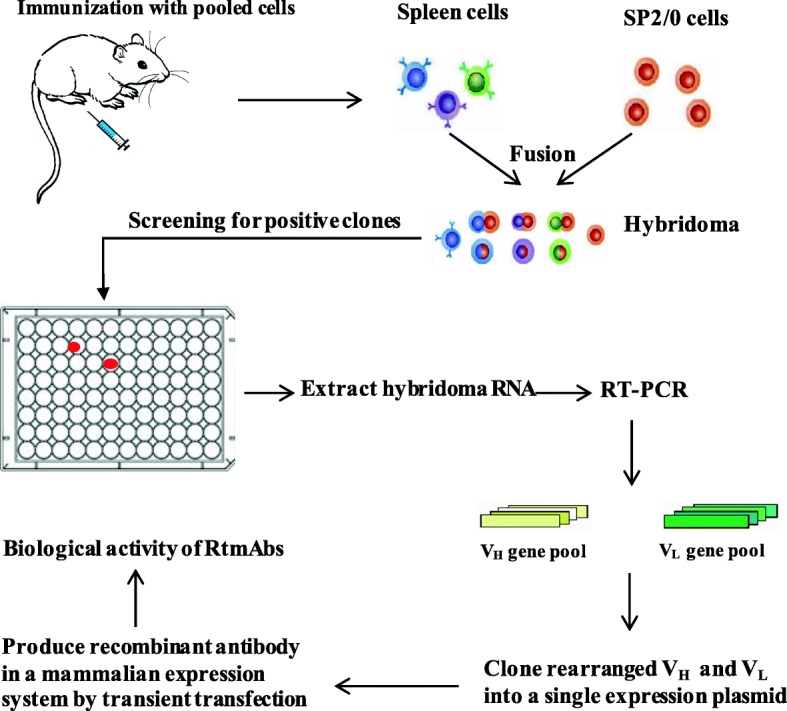
The schematic illustration of a rapid high-throughput method to select recombinant RtmAbs. Rats were immunized with pooled cells consisting of different antigen-expressing cells. Hybridomas were generated by using rat spleen-mouse myeloma fusions, and supernatants were screened for positive clones using indirect cell based ELISA. The rearranged heavy and light chain variable regions were amplified from RNA extracted from hybridoma cells by RT-PCR and cloned into a single expression plasmid. The recombinant RtmAbs were produced by transiently transfecting mammalian cells

The schematic illustration of the method is outlined in Fig. 1. This method is based on cell immunization with pooled cells consisting of antigen-expressing cells and standard hybridoma technology with rat spleen-mouse myeloma fusions, which enable researchers to gain easy access to the immunogen and ensures that the fusion process for hybridoma preparation is operating at high efficiency. We can select RtmAbs against different antigens from a single rat immunized with pooled cells consisting of different antigen-expressing cells, either cell surface antigens or secreted antigens. Because of the time-consuming and labour-intensive process of clonal selection that is required for isolation of stable and homogenous hybridoma clones, positive hybridoma clones were first screened through indirect cell-based ELISA and then directly subjected to RT-PCR for amplifying rearranged heavy and light variable regions, which greatly improved the screening efficiency. Then, the amplified heavy and light variable regions were cloned into a single expression plasmid that could express full-length rat antibodies from a single ORF joined by the FMDV 2A self-processing peptide [15]. Thus, a large number of antibodies can be quickly produced by transiently transfecting mammalian cells. The biological activity of recombinant RtmAbs were thereafter validated in vitro and in vivo by functional evaluation.

### Immunogen design and verification

**Fig. 2:**
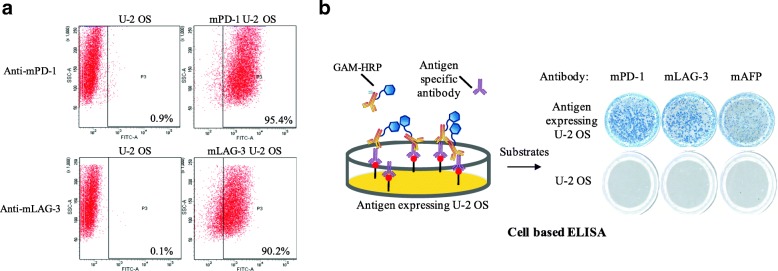
Immunogen design and verification. **a** Cell lines expressing various antigens were established as the immunogens. mPD-1- and mLAG-3-expressing U-2 OS cells were established by lentivirus transduction and were verified by flow cytometry with the indicated antibodies. **b** Cell-based ELISA was established to verify the mPD-1-, mLAG-3- and mAFP-expressing U-2 OS cells. The mPD-1-, mLAG-3- and mAFP-expressing U-2 OS cells were fixed and stained with the indicated antibody and thereafter visualized by Immunospot Analyser

Mouse PD-1-, LAG-3-, and AFP-expressing U-2 OS cells (mPD-1 U-2 OS, mLAG-3 U-2 OS and mAFP U-2 OS) were established separately and verified for their antigen expression. Cell surface antigen mPD-1 and mLAG-3 were analysed by flow cytometry, which showed high expression of the corresponding antigen (Fig. 2a). Because mAFP is a secreted protein, cell-based ELISA was established for verifying antigen expression. After specific antibody staining, mPD-1 U-2 OS, mLAG-3 U-2 OS and mAFP U-2 OS showed high expression of the corresponding antigen (Fig. 2b).

### Proof of principle: Cloning of the 32D6 RtmAb against mouse PD-1

To highly efficiently clone the heavy and light chain variable region of the RtmAb from hybridoma cells, we designed two sets of specific primers to separately amplify the rat immunoglobulin variable regions of the heavy and light κ chain by analysing 115 signal peptide sequences of the heavy chain and 21 signal peptide sequences of the light κ chain retrieved from the IMGT database, which represents 99 and 100% of the signal peptide sequence of the retrieved light and heavy chain. We also designed one universal reverse primer corresponding to the constant region of the heavy chain or the light κ chain, which additionally included a specific sequencing primer for subsequent sequencing (Table 1). Primers to amplify the λ light chains were not included in this study due to a lack of enough signal peptide sequences of the λ chain retrieved from the IMGT database.

**Table 1:** Primers used for the generation of recombinant rat monoclonal antibodies

Reverse primer used for the amplification of heavy chain variable regions	Reverse primer used for the amplification of κ light chain variable regions
R1: AGGATCCAGGRRCCARKGGATAGACAGATG	R2: AGGAGATGGTGGGAAGATGGATACAGTTG
Degenerate primer used for the amplification of heavy chain variable regions	Degenerate primer used for the amplification of κ light chain variable regions
hF:	κF:
1. GCCCGTTTCTGCTAGC ATGTTGGTSCTGMAGTKGGTTTTSGTG	1. GCAATCCAGGTCCA ATGAAAATGACGACACCTGCTCAGTTC
2. GCCCGTTTCTGCTAGC ATGAGACTACTAGGTCTTCTCCTGTGC	2. GCAATCCAGGTCCA ATGAGGGCCCCTTTTCAGTTACTTGGG
3. GCCCGTTTCTGCTAGC ATGGGATGGAGCCAGATCATCCTCTTT	3. GCAATCCAGGTCCA ATGTCTAAAAACTTATTAGAAGTTTCA
4. GCCCGTTTCTGCTAGC ATGGTTTTCGGGCTGATCCTTTTTCTT	4. GCAATCCAGGTCCA ATGGTAAGTCCTGCCCAGTTCCTGTTT
5. GCCCGTTTCTGCTAGC ATGATGGGTTTAGGGGTGATTCTTTTT	5. GCAATCCAGGTCCA ATGATGGCTGCAGTTCAACTCTTAGGG
6. GCCCGTTTCTGCTAGC ATGGGATTGAGCTGGGTTTTTCTTGTT	6. GCAATCCAGGTCCA ATGAAAGTGCCTGGTAGGCTGCTGGTG
7. GCCCGTTTCTGCTAGC ATGGACTTGCGACTGACTTATGTCTTT	7. GCAATCCAGGTCCA ATGAAGTGGCTGTTAGTCTGTTGGTGC
8. GCCCGTTTCTGCTAGC ATGACTATCCTGGTGCTTCTCCTCTGT	8. GCAATCCAGGTCCA ATGATTCCTGCCCAGTTCCTGTTTCTG
9. GCCCGTTTCTGCTAGC ATGGCTRTCCTGGTGCTGTTGCTCTGC	9. GCAATCCAGGTCCA ATGATGAGTCCTGCCCAGTTCCTGTTT
10. GCCCGTTTCTGCTAGC ATGRAATGSARCTGGRTCWTYYTCTTY	10. GCAATCCAGGTCCA ATGAATTTTCAGGTGCAGGTTTTTAGC
11. GCCCGTTTCTGCTAGC ATGGCTGTCCTGGTGCTGTTGCTCTGC	11. GCAATCCAGGTCCA ATGAAYGTGYCCACTCAACTCCTTGGG
12. GCCCGTTTCTGCTAGC ATGAGARYGYYGRKTCTTCTGTACCTG	12. GCAATCCAGGTCCA ATGGACATGAGGGCCCATRCTCAGTTT
13. GCCCGTTTCTGCTAGC ATGGACWTCAGNCTCAGCTTGGBTTTC	13. GCAATCCAGGTCCA ATGARRKTYSNBVYTSAGYTTYKKGGG
14. GCCCGTTTCTGCTAGC ATGRAGTTGDGSMTRANCTGGRTTTTY	
Primer used for the amplification of heavy and light chains	
F3: CATCTGTCTATCCACTGGCTCCTGGAAC	R3: TGACAGGTGCGCGTTTAGCACG TCATTTACCCGGAGAGTGGGAGAG
F4: CGTGCTAAACGCGCACCTGTCAAAC	R4: TGGACCTGGATTGCTTTCTACATC
F5: CAACTGTATCCATCTTCCCACCATC	R5: GATCCGGCCTTGCCGGC CCTAACACTCATTCCTGTTGAAG

The primers used to amplify the heavy and κ light chain were designed using the genome sequence of the rat immunoglobulin: GenBank: DQ402471.1 for the κ light chain and GenBank: BC095846.1 for the heavy chain. Degenerate base code: M = A + C; R = A + G; W = A + T; S = C + G; Y = C + T; K = G + T; V = A + C + G; B = C + G + T; D = G + A + T; N = C + G + A + T

**Fig. 3:**
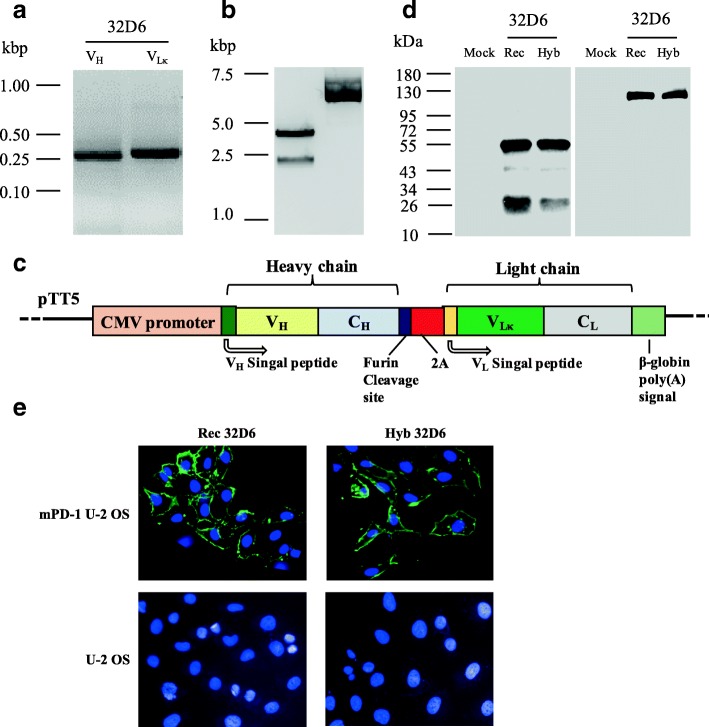
Cloning of the 32D6 monoclonal antibody against mouse PD-1. **a** RT-PCR amplification of the light chain variable region (VLκ) and heavy chain variable region (VH) from the hybridoma 32D6. **b** Assembly of the 32D6 VH region and VLκ region through a joining fragment resulting in a 6.574-kbp product. **c** Schematic of the plasmid encoding the rat recombinant antibody with the FMDV 2A sequence. **d** Western blot analysis under reducing (left panel) or non-reducing (right panel) conditions: fresh culture medium (Mock), supernatant of cells producing the recombinant 32D6 antibody (Rec 32D6) or the 32D6 monoclonal antibody purified from the parental hybridoma (Hyb 32D6). **e** Immunofluorescence analysis of mPD-1 U-2 OS and U-2 OS cells with Rec 32D6 and Hyb 32D6

By using this method, we cloned the heavy and light chain variable regions of the RtmAbs against mouse PD-1, LAG-3 and AFP. We used a hybridoma (32D6) that secreted a rat anti-PD-1 IgG_2a_ as a proof-of-principle. By using the designed primers, we successfully amplified PCR products from the heavy and light chain variable regions with the expected sizes (Fig. 3a). The expression sequence of the heavy chain or κ light chain of recombinant 32D6 contained a signal peptide, a variable region and a constant region. After sequencing confirmation, the κ light chain and heavy chain with their signal peptides were cloned into an expression plasmid PTT5 driven by the CMV promoter, and containing a joint fragment comprising the furin cleavage site and the 2A sequence. The assembly result in a 6.574-kbp product (Fig. 3b and c).

After transient transfection in HEK 293 cells, the plasmid expressed the recombinant rat anti-PD-1 IgG_2a_ antibody. The supernatant of HEK 293 cells producing the recombinant 32D6 antibody (Rec 32D6) was separated by SDS-PAGE gel and analysed by western blot. The results under reducing conditions showed that the recombinant antibody expression from the expression plasmid had a balanced expression level of the heavy and light chains. Under non-reducing conditions, Rec 32D6 appeared as a single band, which indicated the natural association of the heavy and light chain of Rec 32D6 (Fig. 3d).

An indirect immunofluorescence assay was used to identify the binding specificity of Rec 32D6 and Hybridoma 32D6 (Hyb 32D6) purified from the parental 32D6 hybridoma cells, and the results showed that both Rec 32D6 and Hyb 32D6 could specifically bind to the mPD-1 U-2 OS cells (Fig. 3e).

### Biological activity of rec 32D6

**Fig. 4:**
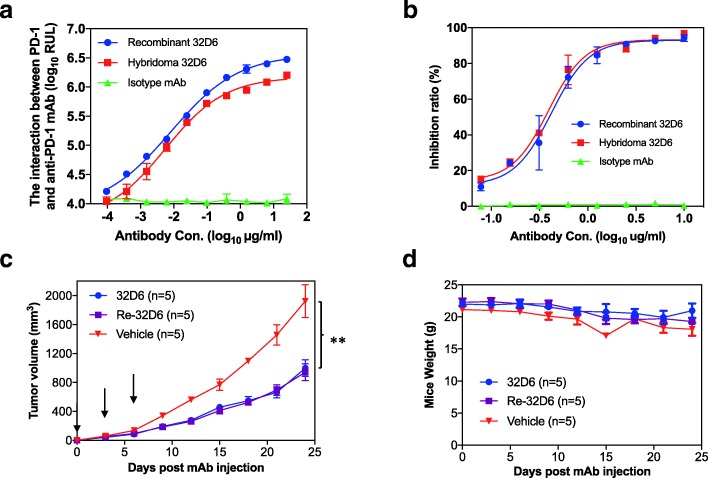
Biological activity of 32D6 RtmAbs. **a** The reactivity of anti-PD-1 (Rec 32D6 and Hyb 32D6) against his-mPD-1 protein was determined by indirect CEIA. **b** The blocking activity of anti-PD-1 (Rec 32D6 and Hyb 32D6) was determined by a blocking CEIA. **c**, **d** The therapeutic efficacy of anti-PD-1 (Rec 32D6 and Hyb 32D6) was evaluated in a syngeneic tumour model bearing a mouse kidney tumour

Rec 32D6 may differ in several respects from Hyb 32D6 secreted by the hybridoma cells. To prove that they have a comparable performance, the biological activity of Rec 32D6 was first evaluated in parallel with Hyb 32D6 in vitro. Using indirect CEIA to measure the binding activity, a good correlation in determining the reactivity of Rec 32D6 and Hyb 32D6 against the mPD-1 protein was observed. Rec 32D6 showed a slightly higher affinity than Hyb 32D6 (Fig. 4a). Using blocking CEIA to measure the blocking activity of Rec 32D6 for interfering with the interaction between mPD-1 and mPD-L1, Rec 32D6 showed a similar blocking activity to Hyb 32D6 (Fig. 4b). The results showed that Rec 32D6 exhibited comparable affinities and full biological activity with Hyb 32D6.

Moreover, we investigated the anti-tumour efficacy of these anti-PD-1 antibodies in a syngeneic tumour model bearing mouse kidney tumours. It was observed that tumour growth was significantly inhibited in both the Rec 32D6 (5/5) and Hyb 32D6 (5/5) treatment groups and there were no toxicities and abnormal weight changes during the treatment (Fig. 4c and d). The results showed that Rec 32D6 exhibited comparable therapeutic efficacy to Hyb 32D6 in vivo.

### Selection of multiple RtmAbs with different IgG isotypes from a single fusion

We immunized rats with pooled cells consisting of three different antigen-expressing cells, and the hybridoma supernatants were tested by high-throughput cell-based ELISA to ensure that the secreted antibodies retained specificity for their antigens. After the fusion process, the hybridoma cells were counted and one eighth of them were plated over twenty 96-well plates at a density of 1 cell/well for culture. After clonal expansion for 2 weeks, 1920 supernatants were screened using cell-based ELISA. Of the three different immunized antigens, 20 positive hybridoma supernatants were identified for these three antigens and each of them were subjected to subsequent recombinant construction. Of these 20, 13 were found to weakly cross-react with U-2 OS cells when re-tested by cell based ELISA, but seven recombinant RtmAbs retained high binding specificity to antigen-expressing U-2 OS cells (Additional file 1: Figure S1). These were subjected to isotype phenotyping (Table 2). For each hybridoma, cDNA was synthesized and used to independently amplify both heavy and light chains before joining them into a single expression plasmid.

**Table 2:** A summary of the antibodies selected from a single fusion

Clone	Antigen	Antibody name	Isotype	VH	VL
1.1	mAFP	10B3	IgG1	V1–68 + D1 + J1	V3S19 + J2
1.1	mAFP	10H3	IgG1	V2–41 + D1 + J2	V3S19 + J2
1.1	mAFP	6B8	IgG2a	V2–43 + D1 + J4	V2S23 + J4
2.1	mPD-1	32D6	IgG2a	V1–28 + D5 + J1	V1S13 + J2
2.1	mPD-1	8E2	IgG2b	V7–7 + D1 + J4	V1S19 + J2
3.1	mLAG-3	12D9	IgG2b	V5–50 + D1 + J2	V12S16 + J2
3.1	mLAG-3	1B2	IgG2b	V5–50 + D4 + J2	V14S14 + J2

**Fig. 5:**
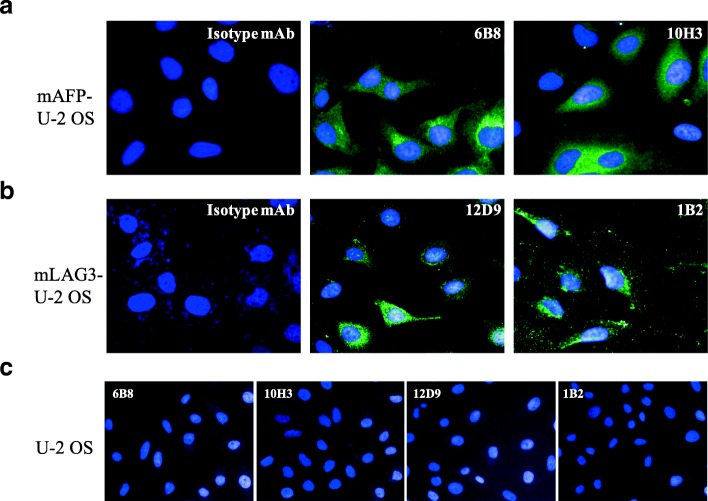
Immunofluorescence analysis of recombinant anti-mLAG-3 and anti-mAFP antibodies. **a** mLAG-3 U-2 OS cells were stained with rat recombinant 12D9 and 1B2 antibodies. **b** mAFP U-2 OS cells were stained with rat recombinant 6B8 and 10H3 antibodies. **c** U-2 OS cells were stained with rat recombinant 12D9, 1B2, 6B8 and 10H3 antibodies

Seven hybridoma cells producing functional antibodies with high specificity for which the heavy and light chains were cloned contained a productively rearranged VH and VL region. For antibody subtyping, two recombinant RtmAbs against mouse AFP belonged to the IgG_1_ isotype, and one recombinant RtmAbs against mouse AFP belonged to the IgG_2a_ isotype. Two recombinant RtmAbs against mouse PD-1 belonged to the IgG_2a_ or IgG_2b_ isotype. Two recombinant RtmAbs against mouse LAG-3 belonged to the IgG_2b_ isotype. All seven RtmAbs were successfully applied in immunofluorescence assays with high binding specificity (Figs. 3e and 5a-c). These data validate that the overall strategy is suitable for high-throughput production of RtmAbs with different IgG isotypes against multiple antigens.

## Discussion

Rat-LOU is excellent for generating mAbs against small and poor immunogenic molecules and yields excellent results against murine antigens. The rat antibodies can be paired with existing antibodies in immunoassays and widely used for therapeutic purposes as cancer immunotherapy.

High-affinity, functionally validated RtmAbs are valuable biomedical reagents, but their acquisition can be time-consuming, labour-intensive and expensive. As far as we know, almost all methods used to produce RtmAbs are based on traditional methods, which are inefficient and inconvenient to obtain the desired RtmAbs. In this study, by immunizing with pooled cells consisting of antigen-expressing cells and quickly cloning the productively rearranged variable antibody regions, we have greatly reduced the time, number of steps, and resources needed to isolate RtmAbs that work in immunotherapy studies and in antigen detection [16]. Importantly, smaller laboratories can benefit from this procedure since only minimal equipment and resources are required.

Compared with traditional methods, our method offers several advantages for producing RtmAbs. The first advantage of this method is cell immunization, which saves not only time and effort but also the amounts of immunogens that are often very valuable or difficult to obtain. We have chosen the injection of whole cells expressing different antigens, which are highly immunogenic and are simple to obtain and use. Although this procedure presents a disadvantage in that hybridomas are produced against virtually all cellular antigens and require several screening steps to select for anti-antigen-specific hybridoma cells, the immunization protocol is easy and function-blocking antibodies against surface antigens (anti-mPD-1 and anti-mLAG-3) and detection antibodies against cellular antigens (anti-mAFP) can both be readily obtained.

Another advantage of this method is that mouse SP2/0 myeloma cells are used as the fusion partner in this method, and there is absolutely no difficulty in preparing rat×mouse hybridomas. Although reliable rat myeloma cells have already been established, there are still some obstacles in their usage, such as poor cellular adherence, low fusion efficiency and low rates of positive rat×rat hybridomas. Therefore, there might be no advantage compared to the rat×mouse hybridoma method. One potential obstacle of the rat×mouse hybridoma might be mandatory use of nude mice or nude rats for obtaining ascites. In our method, the step involving ascites production is eliminated since the antibody expression sequences of desired antibodies are directly amplified from the positive hybridoma clones.

The third advantage is that we can clone the rat antibody genes out of a positive rat hybridoma clone and use our transient transfection technology to produce the rat antibody for use at any scale [17, 18]. Several molecular cloning steps have been eliminated to significantly enhance precise cloning of the positive hybridoma clones using a well-designed set of degenerate primers and the Gibson assembly method.

A few steps that could use improvement were noted during the optimization process. Given that pooled primers were used, it is possible that nonspecific amplification occurred during the PCR process. To minimize this issue, a specific sequencing primer was added to the 5′ terminus of a set of degenerate forward primers used for the amplification of the heavy and light chain variable regions to correct the obtained sequence by sequencing the PCR products. To streamline the molecular cloning process, multiple ligation and digestion steps were eliminated by utilizing the highly efficient Gibson assembly method. Five PCR fragments, including heavy and light chain variable regions, a joint fragment comprising the furin cleavage site and 2A sequence and the heavy and light chain constant regions, were sequentially cloned into a single vector by homologous recombination.

Because therapeutic human mAbs do not always cross-react with animal tissues, experiments in mice models can be performed using a surrogate antibody [19], which is a mAb directed against the murine antigen. Several surrogate mAbs recognizing mouse antigens have been developed against mouse PD-1 [20] or PD-L1 targets in rats [21], which can be considered as surrogates of Keytruda, Opdivo or Tecentriq. Therefore, we shall detail the procedure we followed to produce and screen RtmAbs against mouse PD-1 that was successful in our hands and that can be adapted for specific therapeutic purposes. Moreover, the RtmAbs recognizing the mouse PD-1 antigen have been chimaerised as rat/mouse mAbs to move closer to the murine system for better immunotherapy [22]. Recent advances in tumour immunology have highlighted the key role of these surrogate antibodies in drug development, raising new hopes for rapid high-throughput production of these valuable reagents [23].

## Conclusions

We have reported the development of a novel approach for biomedical scientists that streamlines the production of RtmAbs recognizing mouse antigens. In this paper, we have used this method to raise antibodies against multiple mouse antigens, but it could also be broadly applied to any species that can raise a humoural immune response in rats. As only minimal equipment is required, this technique can also be used by smaller laboratories. Cloning of the rearranged light and heavy chains of selected monoclonal antibodies into a single mammalian expression vector facilitates their distribution, increasing the scope for the creation of valuable antibody resources.

## Methods

### Cells

FreeStyle 293-F cells (catalogue number: R790–07) were purchased from Invitrogen and maintained in FreeStyle™ 293 Expression Medium containing 10% foetal bovine serum (FBS), 100 U/mL penicillin, and 100 μg/mL streptomycin. Human U-2 OS cells (catalogue number: HTB-96), 293 T cells (catalogue number: CRL-3216) and Renca cells (catalogue number: CRL-2947) were purchased from American Type Culture Collection (ATCC) and maintained in Dulbecco’s-modified Eagle’s medium (DMEM) containing 10% foetal bovine serum (FBS), 100 U/mL penicillin, and 100 μg/mL streptomycin.

### Immunogen preparation

The full-length genes of mouse antigens PD-1, LAG-3, and AFP were amplified and ligated into the pLv vector by the Gibson assembly method. The expression vector for each antigen, together with its packaging plasmids, was transfected into 293 T cells using PEI transfection reagents. Supernatants containing lentivirus were harvested 2 days after transfection, and the U-2 OS cells were transduced with the harvested supernatants in the presence of 6 μg/mL polybrene. Then, 24 h after transduction, the U-2 OS cells were treated with 1 μg/mL puromycin for one week. Mouse PD-1-, LAG-3- and AFP-expressing U-2 OS cells were maintained in DMEM containing 10% FBS and 0.5 μg/mL puromycin. Mouse PD-1- and LAG-3-expressing U-2 OS cells were verified by flow cytometry using commercialized antibodies. Mouse PD-1-, LAG-3- and AFP-expressing U-2 OS cells were verified by cell-based ELISA as previously described [24].

### Immunizations

For the preparation of rat monoclonal antibodies, one six-week-old female LOU rat was maintained in individually ventilated cages under specific pathogen free condition and immunized with immunogens at multiple sites by standard vaccination procedures. In brief, the rat received primary immunization with 1 × 10^6^ pooled cells intraperitoneally, consisting of the same amount of PD-1-, LAG-3-, and AFP-expressing U-2 OS cells, and then received immunization (three times) with 6 × 10^6^ pooled cells at two-week intervals. Rats with high serum titres against the indicated immunogens were finally boosted by intra-spleen immunization with 1 × 10^6^ pooled cells 3 days before the hybridoma generation. The use of the rat was approved by the Institutional Animal Care and Use Committee at Xiamen University.

### Hybridoma generation

The hybridoma generation procedure followed the protocol reported previously with minor modifications [25]. The immunized rat was firstly euthanized with pentobarbital (200 mg/kg, i.p.), and then followed by spleen dissection and dissociation. The rat splenocytes and mouse SP2/0 myeloma cells were fused at a ratio of 5:1 using PEG 1450 according to a standard protocol. One eighth of the resulting hybridoma cells were plated on twenty 96-well plates at a density of 1 cell/well and initially grown in RPMI 1640 medium supplemented with 20% foetal bovine serum for 24 h. Then, the hybridoma cells were selected with HAT conditioned medium for 2 weeks.

### Antibody screening

The supernatant from each well was assayed by cell-based ELISA plated with the indicated immunogens. Briefly, 96-well plates were plated with 10^4^ cells/well of a mixed cell pools of three antigen-expressing U-2 OS cells or U-2 OS cells, and the cells were fixed with 1% paraformaldehyde and permeabilized by 0.1% Triton X-100. After washing, the nonspecific binding was blocked with PBS containing 5% BSA, and 50 μl of supernatant was added to the wells for 60 min, followed by washing and reaction with HRP goat anti-rat IgG antibody (Biolegend, San Diego, USA). After the addition of TMB substrate solution for 30 min, the reaction was terminated by addition of an acidic STOP solution. The plates were spin dried and counted using ImmunSpot@S5 UV Analyzer (Cellular Technology Limited). Finally, 20 positive clones secreting antibodies specifically against the indicated immunogens were screened and selected. The isotype phenotyping of antibodies were performed by cell-based ELISA using isotype-specific antibodies. Briefly, after cells plating and fixation, 50 μl of supernatant was added to the wells for 60 min, followed by washing and reaction with HRP goat anti-rat IgM, G1, G2a, G2b and G2c secondary antibody (Invitrogen).

### 32D6 Colonized culture

For stable and homogenous clonal selection, dilution of 32D6 positive cells to 1 cell/ per well to maximize the proportion of wells that contain one single clone. After colonized culture for 2 weeks, the supernatants were assayed for their reactivity with corresponding antigen. A single clone with high reactivity to PD-1 was picked out for further clonal selection. Three or more cloning procedures were carried out until > 90% of the wells containing single clones are positive for antibody production.

### RNA isolation and gene amplification

Twenty positive hybridoma clones were selected for RNA isolation. Total RNA was extracted with TRIzol Reagent (Invitrogen) and was reverse-transcribed into cDNA with random primers by SuperScript® III Reverse Transcriptase (Invitrogen). The cDNA products were then used as the template in PCR amplification with KOD-Plus polymerase (TOYOBO, Japan). The primer sequences designed for amplifying the variable regions of the heavy and light chains are listed in Table 1. The functional rearranged heavy variable region of the positive hybridoma clone was amplified using a set of 14 degenerate forward primers, representing almost all of the signal peptide sequences of the heavy chain, and one universal reverse primer corresponding to the constant region of the heavy chain. Similarly, the functional rearranged light variable region was amplified using a set of 13 degenerate forward primers, representing almost all of the signal peptide sequences of κ light chain, and one universal reverse primer corresponding to the constant region of the κ light chain. The PCR products were separated on a 2% agarose gel. The PCR fragments with approximately 350 bp for the heavy variable region and approximately 320 bp for the light variable region were isolated for further analysis.

### Construction of the antibody expression plasmid

Assembly of the rearranged heavy and light variable regions through a joint fragment composed of the constant region of the heavy chain, furin cleavage site and 2A sequence, as well as the constant region of the light chain, was created by the Gibson assembly method with the primers listed in Table 1. The heavy and light variable regions were amplified from the selected cDNA products with forward primer hF1~hF14 with a leading sequence of the heavy chain containing a NheI site and reverse primer R1, and forward primer κF1~ κF13 and reverse primer R2 containing a NaeI site. The constant region of the heavy chain was amplified from rat genomic DNA with primers F3 and R3. The fragments containing the furin cleavage site and 2A sequence were synthesized and amplified with primers F4 and R4. The constant region of the light chain was amplified from rat genomic DNA with primers F5 and R5. All PCR products were gel-purified and assembled into a NheI and NaeI digested PTT5 vector.

### Antibody production

For antibody production from hybridomas, the hybridoma cells were cultured in EX-CELL® 620 HSF Serum-Free Medium (Invitrogen), and the supernatants were collected five days after culture. For recombinant antibody production, the antibody expression plasmid was transfected into FreeStyle 293-F cells using PEI transfection reagents. Supernatants were harvested 5 days after transfection, filtered, concentrated and stored at 4 °C. mAbs were purified by using protein G chromatography according to the manufacturer’s instructions. Purified anti-mAFP antibodies (10B3, 10H3 and 6B8), anti-PD-1 antibodies (32D6 and 8E2) and anti-LAG-3 antibodies (12D9 and 1B2) were quantified with a BCA assay and stored at − 20 °C at a concentration of around 0.5 mg/mL in PBS.

### Western blotting

mAbs were separated by SDS-PAGE under both reducing and non-reducing conditions and transferred onto a nitrocellulose membrane. After membranes were blocked with 5% BSA for 1 h, they were probed with HRP goat anti-rat IgG antibody (1 μg/mL) overnight at 4 °C, followed by rigorous washing, and finally visualized with the Lumi-Light^PLUS^ western blotting substrate (Roche).

### Immunostaining

Immunofluorescence staining was performed as previously described [24]. In brief, antigen-expressing U-2 OS cells on glass slides were fixed with 1% paraformaldehyde and permeabilized by 0.1% Triton X-100. After blocked with 5% normal BSA in PBS for 1 h, the slides were stained with antigen-specific antibody (1 μg/mL) overnight at 4 °C, then incubated with FITC conjugated secondary antibody before examination using a fluorescence microscope. The enzyme-linked Immunospot Assay was performed by the same procedure, except for using HRP conjugated secondary antibody before examination using the ImmunSpot@S5 UV Analyzer.

### Functional analysis of rat monoclonal antibodies against PD-1

The reactivity of anti-PD-1 RtmAb (32D6) against his-mPD-1 protein was determined by indirect chemiluminescence immunoassay (CEIA). Briefly, 96-well plates were coated with 50 ng/well of his-mPD-1 protein, and nonspecific binding was blocked with PBS containing 20% CBS. Purified hybridoma 32D6 antibody or recombinant 32D6 antibody was first diluted starting from 25 μg/ml in PBS containing 5% BSA, followed by four-fold serial dilutions with 10 gradients. A 100 μl dilution was added to the wells for 60 min, followed by washing and reaction with HRP goat anti-rat IgG antibody (Biolegend, San Diego, USA). After the addition of Luminol solution for 5 min, the plates were measured with a chemiluminescence reader (Berthod, DE). Control rat isotype IgG added at the same concentration served as a control.

To determine the blocking activity of the 32D6 antibody, a blocking CEIA detecting the interaction between his-PD-1 and biotinylated PD-L1 was developed. Briefly, 96-well plates were coated with 50 ng/well of his-PD-1 protein, and nonspecific binding was blocked with PBS containing 20% CBS. Purified hybridoma 32D6 antibody or recombinant 32D6 antibody was first diluted from 10 μg/ml in PBS containing 5% BSA, followed by two-fold serial dilutions with 8 gradients. Then, a 100 μl dilution or PBS and 100 ng/well biotinylated PD-L1 was added to the wells for 60 min, followed by washing and reaction with HRP goat anti-rat IgG antibody (Biolegend, San Diego, USA). After the addition of Luminol solution for 5 min, the plates were measured with a chemiluminescence reader (Berthod, DE). Control rat isotype IgG added at the same concentration served as a control. The inhibitory ratio was calculated as follows: %inhibitory = 100 × (1 − (average value for each dilution/average value for control)). Each dilution was repeated in triplicate wells and each test was carried out in duplicate or triplicate. The results were interpreted by nonlinear, dose-response regression analysis using GraphPad Prism software.

### Animal experiments

Fifteen 6-week-old C57BL/6 female mice were purchased from the Shanghai Slack Laboratory Animal Co., Ltd. and were bred in the Experimental Animal Center of Xiamen University. All mice were maintained in individually ventilated cages under specific pathogen free condition. An inoculum of 10^6^ Renca tumour cells was injected s.c. into the flank of mice in 50 μl sterile PBS. Mice were randomized into two treatment groups and one control group (*n* = 5 for each group) on day 8 following tumour inoculation, immediately before treatment. Purified hybridoma 32D6 antibody or recombinant 32D6 antibody was administered via intraperitoneal injection at 200 μg every three days for a consecutive three dosages in total. Tumour growth and body weight were monitored every three days. Eighteen days after the last treatment, mice were euthanized with pentobarbital (200 mg/kg, i.p.). Tumour size was calculated as (length×width^2^)/2, and the significant of the difference was analysed between different groups using Fisher’s exact test. The use of the mice was approved by the Institutional Animal Care and Use Committee at Xiamen University.

## Additional file

Additional file 1: **Figure S1.** Binding specificity of 20 recombinant RtmAbs. (PDF 940 kb)

## Abbreviations

AFP: Alpha-fetoprotein
CEIA: Chemiluminescence immunoassay
ELISA: Enzyme-linked immunosorbent assay
IgG: Immunoglobulin G
LAG-3: Lymphocyte-activation gene 3
mAb: Monoclonal antibody
PCR: Polymerase chain reaction
PD-1: Programmed cell death-1
PD-L1: Programmed death-ligand 1
RtmAb: Rat monoclonal antibody
RT-PCR: Reverse transcription PCR
