# Supplementary figures and images for: A novel approach for rapid high-throughput selection of recombinant functional rat monoclonal antibodies
Source: BMC Immunol. 2018 Dec 4;19:35. doi: 10.1186/s12865-018-0274-8 (PMC6280491; doi:10.1186/s12865-018-0274-8)

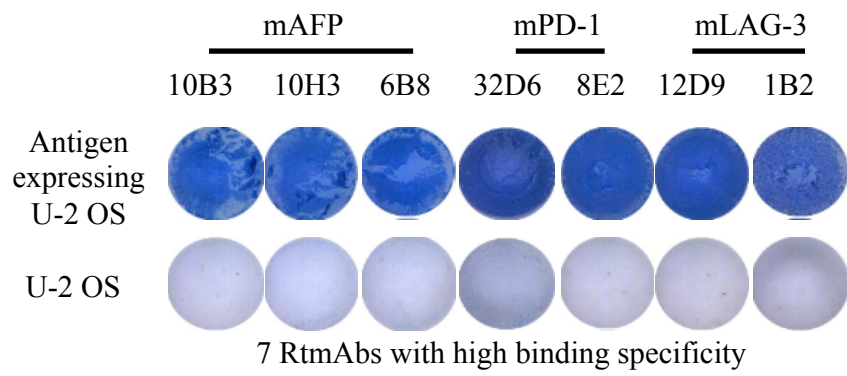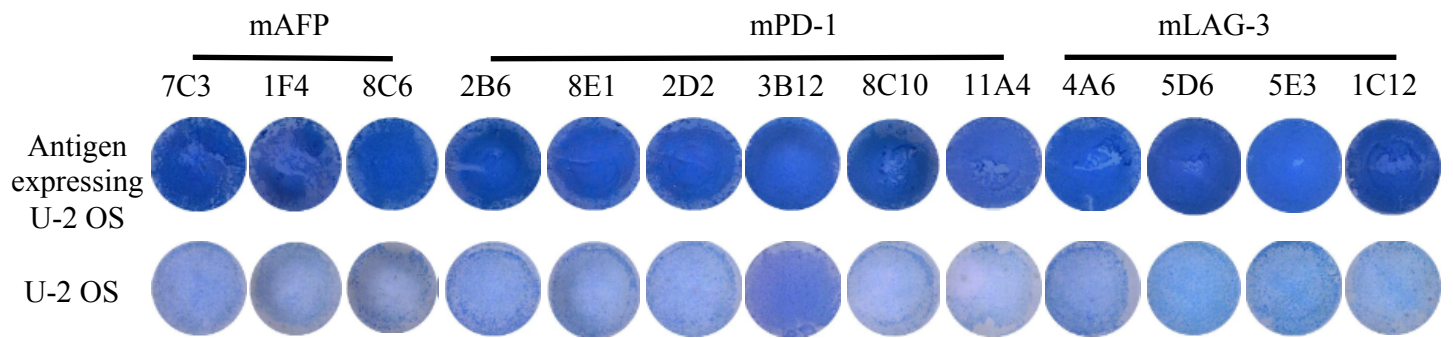

Supplement: Supplementary file 1 — Figure S1. Binding specificity of 20 recombinant RtmAbs. (PDF 940 kb) [file 12865_2018_274_MOESM1_ESM.pdf]
